# Thymol Affects Congruency Between Olfactory and Gustatory Stimuli in Bees

**DOI:** 10.1038/s41598-019-43614-8

**Published:** 2019-05-23

**Authors:** Clara Chapuy, Lisa Ribbens, Michel Renou, Matthieu Dacher, Catherine Armengaud

**Affiliations:** 1 Sorbonne Université, INRA, CNRS, IRD, UPEC, Univ. P7, Institut d’Ecologie et des Sciences de l’Environnement de Paris (iEES-Paris), Paris, France; 2 Centre de Recherches sur la Cognition Animale (CRCA), Centre de Biologie Intégrative (CBI), Université de Toulouse, CNRS, UPS, Toulouse, France

**Keywords:** Classical conditioning, Behavioural ecology

## Abstract

Honey bees learn to associate sugars with odorants in controlled laboratory conditions and during foraging. The memory of these associations can be impaired after exposure to contaminants such as pesticides. The sub-lethal effects of acaricides such as 5-methyl-2-(propan-2-yl)-phenol (thymol) introduced into colonies to control varroa mites are of particular concern to beekeeping, due to detrimental effects of some acaricides on bees. Here we assess whether various odorant/sugar pairs are identically memorized in a differential appetitive olfactory conditioning experiment and whether this learning is affected by thymol exposure. Responses to odorants in retrieval tests varied according to the sugar they were paired with, a property called congruency. Interestingly, congruency was altered by pre-exposure to some thymol concentrations during retrieval tests, although electroantennography recordings showed it left odorant detection intact. This highlights the importance of taking into account subtle effects such as odor/sugar congruency in the study of the effect of pesticides on non-target insects, in addition to the simpler question of memory impairment.

## Introduction

Among the factors implicated in the decline of honey bees (*Apis mellifera*, Hymenoptera, Apidae) particularly observed in North America and Europe, chemical agents are in the foreground because of their multiple effects on learning^1,2^, sensory abilities^3^ and foraging^4,5^. Olfactory conditioning is an efficient tool to explore whether the alteration of the neurophysiological processes underlying bee learning and memory^6–10^ by exposure to pesticides^2,3,11^ is involved in this decline. This is particularly relevant to the pesticides that interfere with γ-aminobutyric acid (GABA) transmission. Among them the acaricide thymol is widely applied inside hives to control infestation with varroa mites (*Varroa destructor*, Acari, Mesostigmata) so that any sub-lethal effect of this intra-colony pesticide is of particular concern for beekeeping^12–15^. We previously reported a loss of specificity of olfactory memory in honey bees following topical application of thymol^16^. This effect on cognition appears similar to those reported for the GABA ligands fipronil^3^ or picrotoxin^17^, and thymol potentiates the GABA response in *Drosophila melanogaster*^18^.

In our previous work, bees were trained to associate a single odorant, either 2-hexanol or 1-nonanol, with sucrose as a reward, following classical conditioning procedures^16^. This work pointed to a thymol effect on specificity of memory, which can be confirmed by using differential conditioning. Indeed, such protocols are specifically designed to evaluate discrimination and generalization of odorants, as animals learn to respond to an odor and to ignore another one^19^. Moreover, *in natura*, honey bees encounter a wide variety of floral bouquets and they feed on nectar, which is mainly composed of sucrose but also contains other sugars such as fructose although in a lower proportion^20,21^. In addition to nectar, fructose is an important food source for bees as it is one of the main sugars in honey^20,22–26^, which in contrast to nectar does not include sucrose.

As a result, we studied the effect of thymol on differential olfactory conditioning. Bees readily learn to associate floral odors with food, and it has been demonstrated that odor/food associations performed while flying freely to natural or artificial sucrose food sources can be transferred to the restrained conditions, and vice-versa^27–30^.

In laboratory conditions tethered bees are often trained to associate alcohols such as 1-hexanol or 1-octanol with a sucrose solution. This type of protocol relies on the proboscis extension response (PER): when a sugar solution touches its antennae, the bee reflexively extends its main mouthpart, the proboscis. The PER can be conditioned with Pavlovian conditioning during which bees associate an odorant or conditioned stimuli (CS) in the terminology of Pavlovian conditioning, and a sucrose reward or unconditioned stimulus (US). Following this training, presentation of the odorant alone becomes sufficient to elicit the PER^31^. Recent reports indicate that the nature of the sugar used as US during training affects the robustness of memory: bees develop little long-term memory when 1-hexanol is associated with fructose^32^ whereas sucrose and glucose led to higher conditioned PER. Moreover, all odorants are not learnt equally well^8,33^. This suggests some odor/sugar combinations are easier to memorize than others because bees have experienced some odorant molecules of floral bouquets with a sweet taste in nectar. The perceived “harmonious” combination, a phenomenon called congruency, helps bees to represent the food in its context. In humans for instance, perceived odor-taste congruence influences intensity and pleasantness^34,35^ as well as memory^36,37^.

The objective of this study was to determine if the effect of thymol on bee’s memory was generalizable to other olfactory and gustatory stimuli combinations^32^ and whether it affected congruency, a parameter not previously taken into account in the study of bee memory. We used either sucrose or fructose and 4 odorants (1-hexanol, 2-hexanol, 1-octanol or 2-octanol) with and without thymol treatment. These odorants are present in floral^38,39^ and bee pheromone compounds^40^. Fructose could be better associated with the odorants present inside the hive (such as pheromone components 1-hexanol, 1-octanol) than sucrose because it is the main sugar of honey and it is less abundant than sucrose in nectars. From this, it could be envisaged that fructose is congruent with the odorants of the colony and sucrose with floral odorants.

## Results

### Thymol does not affect odor detection

**Figure 1:**
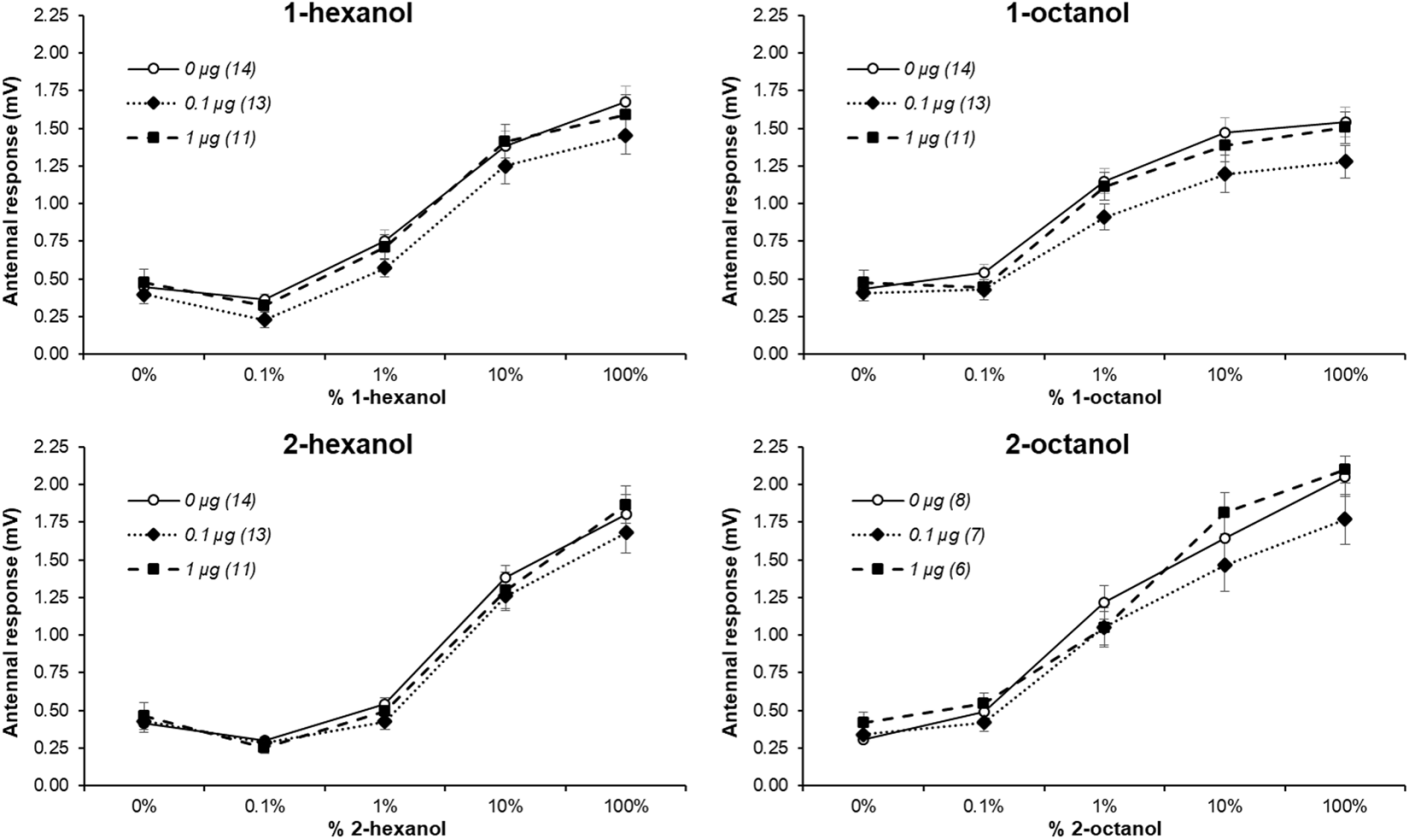
Effect of thymol on the bee olfaction. Electroantennogram responses in mV (mean with standard error) for increasing concentrations of 1-hexanol, 2-hexanol, 1-octanol and 2-octanol diluted in mineral oil after exposure to thymol. Each curve corresponds to a pre-exposure to a thymol concentration; values in parenthesis are the sample size (the same animals were used but some were not tested with 2-octanol).

First, as a control experiment, we checked whether thymol affects odorant detection. To do so, 1 µl of a thymol solution dissolved in 1% ethanol was applied topically on the dorsal thorax 3 hours before the recordings (11 bees received 1 µg/µl, 13 bees received 0.1 µg/µl and 14 control bees received 0 µg/µl). We recorded electroantennograms to establish dose-response curves for each of the four odorants used later (1-hexanol, 2-hexanol, 1-octanol and 2-octanol diluted in mineral oil at concentrations of 0%, 0.1%, 1%, 10% and 100%). Electrophysiological recordings of the antennae revealed an expected significant concentration-dependent increase of the response for each of the four odors (Fig. 1, repeated measurement in a mixed model: ps < 0.0001 for the concentration effect in the four models). Conversely, there was no effect of thymol treatment or interaction between thymol treatment and odor concentration (mixed model, ps ≥ 0.175 for the thymol effect and the interaction thymol*concentration in the four models). This indicates that thymol does not affect odorant detection at the antenna level.

### Performance during training

Other thymol-treated bees were submitted to a differential conditioning protocol during which an odorant was paired with a sugar (CS+) whereas another odorant was not (CS−). This protocol assesses whether animals are able to discriminate between the two odorants. Two pairs of odorants were used: 1-hexanol vs 1-octanol, and 2-hexanol vs 2-octanol (see details of groups in Table 1). The sugar paired with the CS+ was either sucrose or fructose. There were 8 learning trials (4 with CS+ and 4 with CS−) in a pseudo-random sequence (see methods). Table 1 reports the sample size in each treatment combination; within a treatment, they vary between training and retrieval tests as some bees died or became unresponsive to sugar. Training data were analyzed with generalized estimating equations (GEE), using the following factors: trial as a repeated measurement (random factor), thymol treatment, sugar and odor. One analysis was done for CS+, and another one for CS−.

**Table 1:** Number of bees of each experimental group.

Odor (CS+/CS− combinaisons)s	Sugar (US)	Thymol treatment	Bee numbers		
			Learning	Test 1 h	Test 24 h
1-hexanol CS+, 1-octanol CS−	Fructose	0 µg	36	36	22
	Sucrose		39	38	24
1-octanol CS+, 1-hexanol CS−	Fructose	0 µg	27	27	20
	Sucrose		23	23	18
2-hexanol CS+, 2-octanol CS−	Fructose	0 µg	37	37	32
	Sucrose		37	37	36
2-octanol CS+, 2-hexanol CS−	Fructose	0 µg	22	22	19
	Sucrose		22	22	19
1-hexanol CS+, 1-octanol CS−	Fructose	0.1 µg	34	34	21
	Sucrose		41	41	23
1-octanol CS+, 1-hexanol CS−	Fructose	0.1 µg	25	25	16
	Sucrose		29	28	19
2-hexanol CS+, 2-octanol CS−	Fructose	0.1 µg	34	34	28
	Sucrose		40	40	35
2-octanol CS+, 2-hexanol CS−	Fructose	0.1 µg	21	21	14
	Sucrose		22	22	19
1-hexanol CS+, 1-octanol CS−	Fructose	1 µg	32	32	21
	Sucrose		42	42	28
1-octanol CS+, 1-hexanol CS−	Fructose	1 µg	26	26	21
	Sucrose		30	30	22
2-hexanol CS+, 2-octanol CS−	Fructose	1 µg	34	33	31
	Sucrose		32	32	28
2-octanol CS+, 2-hexanol CS−	Fructose	1 µg	20	20	19
	Sucrose		20	20	14

For each experiment, bees were pre-treated with one dose of thymol (0, 0.1 or 1 µg). Then they were conditioned with one of four odorants (1-hexanol, 1-octanol, 2-hexanol or 2-octanol; CS+), paired to either fructose or sucrose as unconditioned stimulus (US). During learning, another odor was used as CS− (see methods). Some bees were lost during the 1 h or 24 h test because they stopped responding to sugar or died.

**Figure 2:**
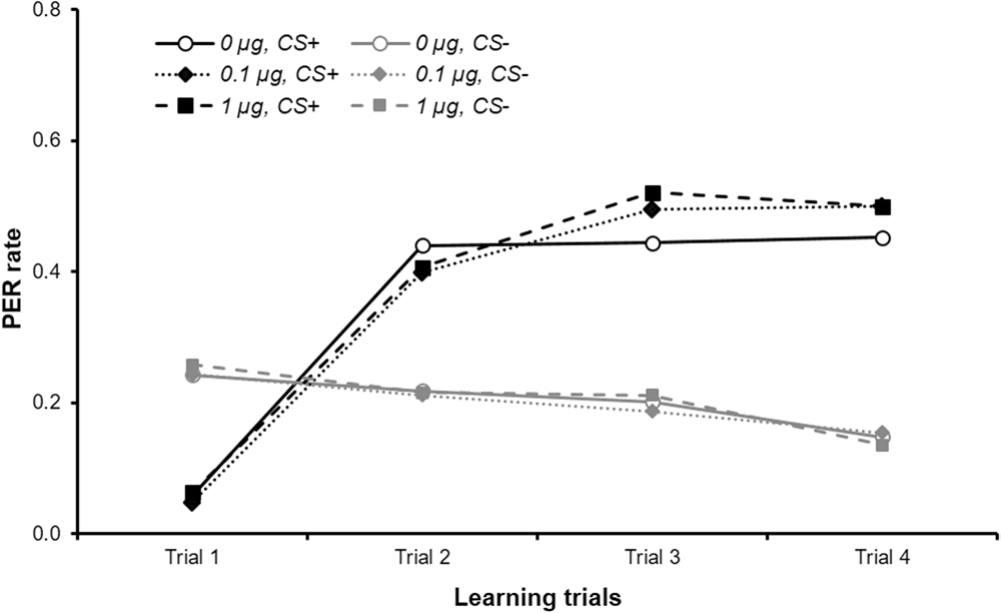
Bee PER rate during the learning trials. Each curve corresponds to the response to the CS+ or the CS− for different thymol treatments. As there was neither significant difference between the different odorants and sugars, nor any interactions, the odorant and sugar groups were pooled. A total of 725 bees were used (20–42 in each combination odorant/sugar/treatment, see Table 1). The initial high response rate to CS− is a usual observation in differential conditioning and corresponds to generalization from CS+.

During training (Fig. 2) the rate of PER performance by bees increased in response to the CS+ (i.e. the trained odor) during the four trials (GEE, trial effect, p < 0.0001) whereas the PER rate in response to the CS− was constant or even slightly decreasing (GEE, trial effect, p = 0.091). This indicates animals learnt to specifically recognize the odorant associated with sugar. For CS+, neither thymol treatment, sugar used, odorant employed nor interactions between these factors or with the trial factor affected the PER rate (GEE: p = 0.059 for 2-hexanol compared to 1-hexanol, p = 0.096 for 2-octanol compared to 1-hexanol, ps ≥ 0.132 for all other odor comparisons, factors thymol and sugar, and all interactions). For CS−, these effects were not significant either (GEE: p = 0.088 for the interaction 2-octanol*thymol 0.1 µg, p = 0.059 for the interaction 2-octanol*thymol 1 µg, ps ≥ 0.102 for all other interactions and for the factors odor, thymol and sugar). As there was no significant interaction between sugar and odorant factors we concluded that there was no difference in congruency between the odorants relatively to the two sugars during olfactory conditioning.

Thus, in spite of the thymol treatment, the bees could undergo Pavlovian conditioning with the same level of PER for all the odorant/sugar combinations tested. Moreover their capacity to discriminate between the two odorants was not affected by thymol.

### Retrieval test 1 hour after training

One hour after training animals were presented again with the CS+ and the CS− without sugar. As shown in Table 2, there was no significant change in the performance between the last learning trial and the retrieval test (McNemar test, adjusted ps ≥ 0.116 for all odor/sugar/thymol combinations); this indicated the performance did not vary between training and retrieval. Performance during the retrieval was then analyzed by comparing PER rate with a logistic regression, using the factors odor, sugar, thymol treatment and their interaction.

**Table 2:** PER rate to CS+ during the last acquisition trial, 1-hour and 24-hour retrieval tests.

Odors	Sugar (US)	Thymol treatment	Trial 4	Test 1 h	Test 24 h
1-hexanol CS+, 1-octanol CS−	Fructose Sucrose	0 µg	0.42	0.42	0.50
			0.44	0.50	0.67
1-octanol CS+, 1-hexanol CS−	Fructose Sucrose	0 µg	0.41	0.41	0.25
			0.57	0.65	0.61
2-hexanol CS+, 2-octanol CS−	Fructose Sucrose	0 µg	0.51	0.35	0.38
			0.49	0.46	0.47
2-octanol CS+, 2-hexanol CS−	Fructose Sucrose	0 µg	0.36	0.32	0.37
			0.41	0.36	0.58
1-hexanol CS+, 1-octanol CS−	Fructose Sucrose	0.1 µg	0.59	0.59	0.71
			0.49	0.54	0.70
1-octanol CS+, 1-hexanol CS−	Fructose Sucrose	0.1 µg	0.56	0.56	0.44
			0.52	0.54	0.68
2-hexanol CS+, 2-octanol CS−	Fructose Sucrose	0.1 µg	0.41	0.41	0.39
			0.58	0.53	0.57
2-octanol CS+, 2-hexanol CS−	Fructose Sucrose	0.1 µg	0.43	0.48	0.36
			0.36	0.36	0.58
1-hexanol CS+, 1-octanol CS−	Fructose Sucrose	1 µg	0.59	0.50	0.48
			0.60	0.69	0.64
1-octanol CS+, 1-hexanol CS−	Fructose Sucrose	1 µg	0.69	0.73	0.57
			0.43	0.40	0.73
2-hexanol CS+, 2-octanol CS−	Fructose Sucrose	1 µg	0.44	0.39	0.42
			0.44	0.34	0.50
2-octanol CS+, 2-hexanol CS−	Fructose Sucrose	1 µg	0.40	0.40	0.42
			0.30	0.30	0.36

**Figure 3:**
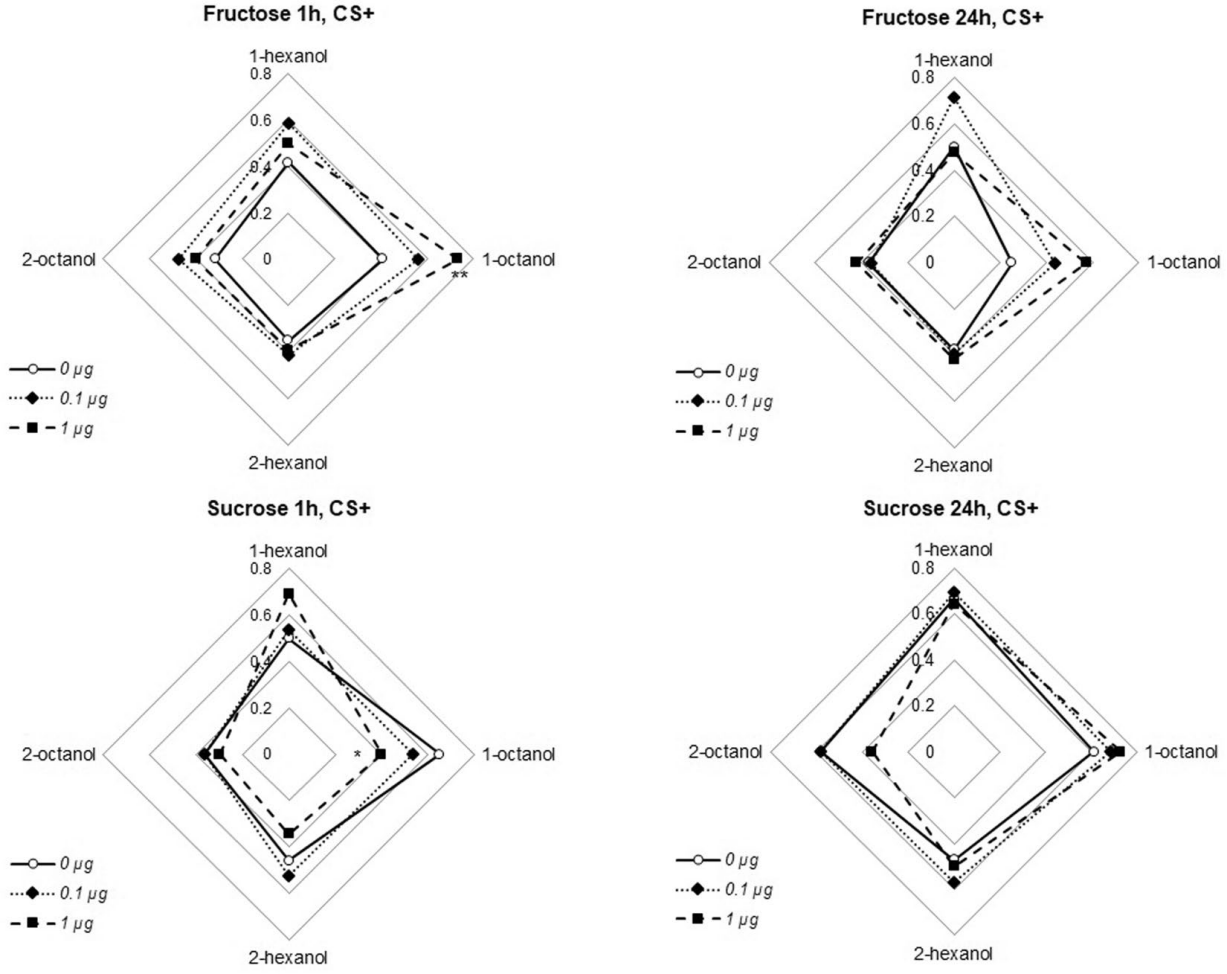
PER rates for the CS+ during the retrieval tests performed 1 hour and 24 hours after the training reported in Fig. 2 (the corresponding CS− data are in Fig. 4). The upper radar plot shows scores of bees trained with fructose the lower plot bees trained with sucrose; plots on the left are for 1-hour retrieval tests, and plots on the right for 24-hour retrieval tests. The curves correspond to the different thymol treatment. The tip of the plots corresponds to the odorants used as CS+. Stars denote significant interaction between 1 µg thymol treatment, fructose and 1-octanol (logistic regression, **p < 0.010) or 1 µg thymol and 1-octanol (logistic regression, *p < 0.050); this kind of interaction is the hallmark of congruency alteration.

Data are the same as in Fig. 2 (Trial 4) and Fig. 3 (Test 1 h and Test 24 h).

During this retrieval test (Fig. 3), the main factors have no significant effects, neither odorant used (logistic regression, comparisons between odors: ps ≥ 0.249), sugar used (logistic regression, comparisons between sugars: p = 0.473) nor thymol doses (logistic regression, comparisons between thymol doses: ps ≥ 0.085). However, there was a significant increase of conditioned responses specifically for 1-octanol with fructose in animals treated with 1 µg thymol (logistic regression, significant interaction 1-octanol * 1 µg thymol * Fructose: p = 0.007). Interestingly the same dose of thymol significantly decreased the response to 1-octanol with sucrose (logistic regression, interaction 1-octanol * 1 µg thymol: p = 0.013). This suggests 1 µg thymol affected congruency level between the sugar used and 1-octanol but not the three other odorants. No other interactions was significant (logistic regression, interaction 2-hexanol*thymol 1 µg, p = 0.059; all other interactions, ps ≥ 0.177).

**Figure 4:**
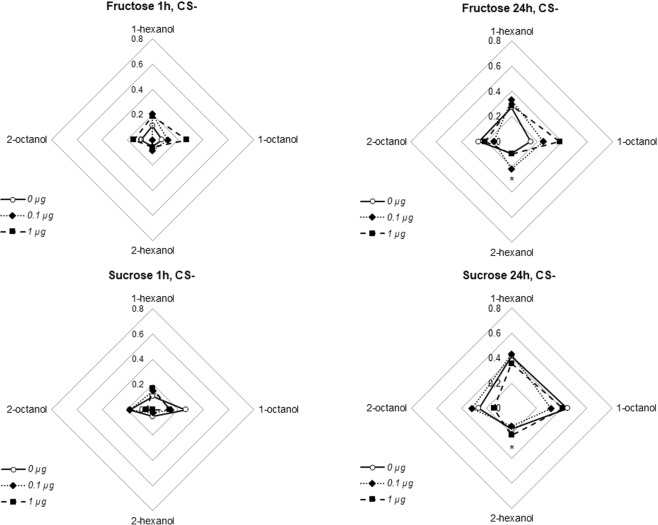
PER rates for the CS− during the retrieval tests performed 1 hour and 24 hours after the training reported in Fig. 2 (the corresponding CS+ data are in Fig. 3). Star denotes a significantly lower PER rate in response to 2-hexanol, irrespective of the sugar used or the thymol treatment (logistic regression, *p < 0.050). Other details are as in Fig. 3.

There was no significant effect of the three factors or their interactions on the response level to the CS− (logistic regression, p ≥ 0.122 for all comparisons and interactions; Fig. 4). Moreover, the PER rate for CS+ was always significantly higher than for CS− (McNemar’s test comparing PER rates to CS+ and CS−: ps ≤ 0.046 in all cases). As expected, animals hardly respond to the CS− during the retrieval test, confirming the specificity of the learning and the absence of generalization; this means that thymol does not increase generalization in these experimental conditions. However, as for CS+, the response rate to CS− seems to depend both on the odor presented as CS− and sugar associated with CS+. In the sucrose group the control response rate was 26% for 1-octanol and only 7% for the same odorant in the control fructose group. Moreover, 1 µg thymol treatment decreased the response to 1-octanol with sucrose as US and increased the response to the same odors when CS+ was paired to fructose. Even though no significant difference was observed (due to the low number of animals responding to CS−), responses to 1-octanol are consistent whether it is used as CS+ or CS−.

### Retrieval test 24 hours after training

One day after training, a new retrieval test was done again as previously. There was no significant change in the PER rate in response to CS+ between the last learning trial and this 24-hour retrieval test (Table 2; McNemar test, adjusted ps ≥ 0.102 for all odor/sugar/thymol combinations), suggesting animals did not forgot the task. Moreover, neither the main effects (odor employed as CS+, thymol treatment or sugar used) nor their interactions were significant (logistic regression, ps ≥ 0.109; Fig. 3).

Similarly, there was no significant effect of any main factors (odor, thymol or sugar) or their interactions on the response level to the CS− (logistic regression, ps ≥ 0.298, Fig. 4), except for a significantly lower PER rate in response to 2-hexanol (logistic regression, p = 0.037).

Even though the response rate was always lower for CS− than for CS+, in some cases the performance was not specific to CS+ in the control group; details and statistics are provided in Table 3. As animals did discriminate during the 1 h retrieval test, this is a case of dissociation between medium-term (1 h) and long-term (24 h) memory trace. Interestingly, 0.1 and 1 µg thymol restored the discrimination (Table 3) for 1-hexanol/fructose and 1-octanol/sucrose, and 1 µg thymol also restored discrimination for 1-octanol/fructose and 2-octanol/fructose but also prevented it for 2-octanol/sucrose. Overall we observed that long-term memory of odorant/sugar associations could be altered by thymol for some odorant/sugar combinations.

**Table 3:** Comparisons between CS+ and CS− for each combination during the 24-hour retrieval test (p-values from McNemar’s test).

Odors	Sugar (US)	Thymol treatment	p-values
1-hexanol CS+, 1-octanol CS-	Fructose	0 µg	0.059°
	Sucrose		0.014*
1-octanol CS+, 1-hexanol CS-	Fructose	0 µg	0.317
	Sucrose		0.083°
2-hexanol CS+, 2-octanol CS-	Fructose	0 µg	0.003*
	Sucrose		0.0009***
2-octanol CS+, 2-hexanol CS-	Fructose	0 µg	0.157
	Sucrose		0.014*
1-hexanol CS+, 1-octanol CS-	Fructose	0.1 µg	0.005**
	Sucrose		0.014*
1-octanol CS+, 1-hexanol CS-	Fructose	0.1 µg	0.083°
	Sucrose		0.008**
2-hexanol CS+, 2-octanol CS-	Fructose	0.1 µg	0.025*
	Sucrose		0.0001***
2-octanol CS+, 2-hexanol CS-	Fructose	0.1 µg	0.083°
	Sucrose		0.025*
1-hexanol CS+, 1-octanol CS-	Fructose	1 µg	0.046*
	Sucrose		0.021*
1-octanol CS+, 1-hexanol CS-	Fructose	1 µg	0.046*
	Sucrose		0.008**
2-hexanol CS+, 2-octanol CS-	Fructose	1 µg	0.002**
	Sucrose		0.005**
2-octanol CS+, 2-hexanol CS-	Fructose	1 µg	0.046*
	Sucrose		0.083°

Data are from Figs 3 and 4. Symbols in the last column highlight statistical significance (°0.100 > p ≥ 0.050; *p < 0.050; **p < 0.010; ***p < 0.001).

## Discussion

When effects of thymol on memory were analyzed through differential olfactory conditioning, they were shown to vary according to the CS+/US combination used in the conditioning paradigm (1-octanol/fructose vs 1-octanol/sucrose in the 1-hour retrieval test). Thus, thymol significantly modified bee performances in 1 h retrieval for specific odorant/sugar pairs only. Moreover during long-term memory recall (24 h retrieval test) thymol sometimes facilitated discrimination and sometimes prevented it (see details in Table 3). By contrast, thymol did not affect odorant detection at the antenna level and performance during learning in differential conditioning was intact. This suggests thymol left odorant perception, sugar sensitivity and PER responsiveness intact. Altogether our results suggest thymol did not alter memory as a whole but instead modified specific memory traces.

During 1 h retrieval, pre-treatment with 1 µg thymol induced a change: response to 1-octanol increased relatively to other odors when it had been previously associated with fructose but decreased when associated with sucrose. These differences were not due to fructose itself, as they were not observed during learning or long-term memory for other odorants. During learning, central neural integration of sugar and the learnt odor alter the representation of the odor so that it acquires a positive valence and triggers an appetent behavior (PER). This integrative process seems modified in bees after thymol exposure for some odor/sugar combination. Thus, we propose that pre-training exposure of 1 µg thymol alters congruency between CS+ and US during memory recall (retrieval test).

In humans, odor-sugar pairs were either congruent or incongruent in the context of feeding behavior, demonstrating the role of experience in odor/taste integration^41^. Odors ranged from highly congruent to highly incongruent with sugar: for example, an odor–induced enhancement of performance is found for a sucrose/strawberry combination but not for the incongruent sucrose/ham^35^ mixture. Response decrease can be induced by conflicts elicited by the occurrence of incongruence in the trial sequences: stimulus conflicts trigger inhibition or avoidance. Similarly, mood-congruent facilitation can occur during retrieval of emotional information^37^.

Variations in stability of associative memory indicate congruency is also present in insects. For instance, Simcock *et al*. reported that bees are good at remembering 1-hexanol associated with sucrose or glucose but not with fructose^32^, indicating that olfactory memory is affected by sugar identity. In our study, control bees had identical responses to 1-hexanol tested at 1 h when it was paired to sucrose or fructose. However, the response to 1-octanol was higher when paired with sucrose than with fructose. Whether an odor/taste combination is congruent depends on familiarity with them or foraging experience before the laboratory test: bees pre-fed with solutions containing an amino acid were less likely to associate odors with sucrose^42^. This would explain the contrast between the present results and Simcok *et al*.’s^32^. Interestingly, the most available sugar is different for winter and summer bees. Honey, the unique food of winter bees, is rich in fructose and glucose. By contrast, foraging summer bees consume nectar, which is rich in sucrose as well as glucose and fructose^20–26^. Thus, it would be particularly interesting to compare their responses. This is all the more relevant that anti-varroa treatments such as thymol are often performed in winter.

To account for the differences between the odorants we used as CS+ we propose that thymol exposure highlighted the potential conflict between sucrose, an appetitive stimulus, and 1-octanol and 1-hexanol that are components of the alarm pheromone^40^. As thymol is a positive allosteric modulator of GABA receptors^18^ we postulate its effects are mediated through GABA circuits. This hypothesis can be tested by looking at the effects of GABA blockers such as fipronil or picrotoxin, on recovery tests after differential conditioning. We have previously reported that after picrotoxin injection in the antennal lobes, bees fail to discriminate between 1-hexanol and 1-octanol when tested for short-term memory^17^. Fipronil makes the bees generalize between 1-hexanol and 1-nonanol 24 h and 48 h after learning^3^. Conversely, in the experiments described in the present paper, control animals failed to discriminate CS+ and CS− during long-term memory tests when 1-hexanol and 1-octanol were paired with fructose, but thymol restored the discrimination. This is fully consistent with our prediction that thymol and picrotoxin should have opposite effects on odorant-sugar associations.

Pesticide impacts in honey bees are not consistent^43^. Currently bees are exposed to fluctuating environmental conditions which combined with internal factors could affect their physiological and behavioral responses to pesticides. Thus, data obtained for contaminants with olfactory conditioning paradigms in bees can be differently interpreted according to subtle differences in the experimental factors. For instance in laboratory conditions learning performances of honey bees are differentially affected by a pesticide according to the learning task: acute treatment with the miticide coumaphos improved short-term memory in massed conditioned bees but not in spaced conditioning^44^. Finally it is noteworthy to mention that the alcohols used as CS are not only floral compounds but also alarm pheromone components increasing recruiting^40^. Thus subtle changes of the congruency in odor-induced taste representation after thymol exposure could significantly impact foraging and intra-hive feeding behaviors in natural conditions.

## Material and Methods

### Chemicals

Thymol solutions were prepared with thymol powder (99.5%, Sigma. CAS 89-83-8) first dissolved in ethanol and then diluted in deionized water. Final concentrations of thymol were 1 µg/µl, 0.1 µg/µl and 0 µg/µl (control) in water-ethanol. The concentrations used were sub-lethal^45^.

Odorants used for olfactory conditioning were 1-hexanol (>99%, Sigma-Aldrich, CAS 111-27-3), 1-octanol (>99.5%. Fluka, CAS 111-87-5), (+−)2-hexanol (>98%. Fluka, CAS 626-93-7) and (+−)2-octanol (97.8%, Sigma, CAS 123-96-6). The sugar solutions used were sucrose 40% w/w (powder, >99.5%, Sigma, CAS 57-50-1) or fructose 40% w/w (powder, 98%, Aldrich, CAS 57-48-7) in deionized water.

### Animals

Bees used in these experiments were *Apis mellifera* (Hymenoptera: Apidae) from the apiary of the Centre de Recherches pour la Cognition Animale in Toulouse (France) or hives in Versailles INRA for electrophysiological experiments. They were fed with their own honey and didn’t receive any treatment except Versailles hives which had received oxalic acid for varroa control the previous winter. PER experiments were performed in 2015 from February to April and from July to September. Electrophysiological recordings were performed between the two PER experiment periods.

Bees were captured before 9:30 AM at the entrance of the hive, cooled in ice until immobile (~5 min) and harnessed in small tubes so that their antennae and proboscis could freely move. After recovering from cooling, they were fed individually with 3 µl of 40% sucrose solution to normalize their feeding motivation and treated with a topical application of 1 µl of one of the thymol solutions (1 µg/µl, 0.1 µg/µl or 0 µg/µl as control) on dorsal thorax. Experiments were conducted more than 3 hours after this treatment to enhance their motivation for sugar; in the meantime they were kept at 28 °C in the dark.

### Electrophysiology

One antenna was immobilized horizontally at the level of the pedicel with dental glue. Electrodes were composed of chlorinated silver wires inserted into glass capillaries filled with physiological solution (NaCl 9 g, KCl 0.2 g, glucose 4.36 g in 1 l of distilled water)^46^. Reference electrode was inserted into the eye and recording electrode capped the tip of the antenna (which was previously cut). The electrodes were connected to an amplifier via a pre-amplifier (NPI electronic). Antennal signals were amplified 1000 times (amplifier ELC-03X, npi electronic), filtered between 0.3 Hz and 500 Hz and digitized at a resolution of 16 bit and 1 kHz sampling rate by a analog-digital conversion card (Data Translation 9800). They were then analysed with MEAD, a dedicated script home-developed with Measure Foundry. The electroantennography set-up was surrounded by a Faraday cage to limit electromagnetic noise.

A main humidified airflow of 70 l/h was directed to the antennae and placed 1 cm away from it to allow the bee to be habituated to airflow mechanical stimulation. The pipette containing the stimulation odorant was connected to a secondary air flow of 10 l/h which was activated during stimulation and added to the main flow. A volume of 5 ml of each odorant solution was deposited on a filter paper in a Pasteur pipette. Fresh stimulus sources were prepared daily before starting experiments and stored until use in a sealed test tube. A new source was used for each bee. An automatic stimulation system (ValveBank. AutoMate Scientific) was used to release a 500 ms odor puff. Each bee was tested with four concentrations (0.1%, 1%, 10%, 100%) of the four odorants diluted in mineral oil as well as control stimulations containing the mineral oil alone. Stimulations were spaced at least 90 s to avoid saturation or sensory adaptation of the antenna stimulated by odorants; exhaustion behind the bee prevented odorant accumulation.

### Olfactory conditioning and memory test

We used a differential conditioning procedure^19,47^. This procedure tested the ability of bees to distinguish between two odorants. Bees were placed in a box with a constant airflow so that any odorant was removed after a trial. The odor stimulations were made with a plastic syringe containing a 1*1 cm square of filter paper with 5 µl of pure odorant; the filter paper was changed every day. Bees were alternately stimulated with two odorants: one odorant (positive conditioned stimulus or CS+) was always associated with the US whereas the second odorant was never associated with the US (negative conditioned stimulus or CS−). To avoid bias caused by the order of appearance we used pseudo-random sequences: either CS+, CS−, CS−, CS+, CS+, CS−, CS−, CS+ or CS−, CS+, CS+, CS−, CS−, CS+, CS+, CS−, with 1 min interval between trials in all cases. Thus four trials were conducted with CS+ and four with CS−. Two pairs of odorant were used as CS+ and CS−: 1-hexanol was paired with 1-octanol and 2-hexanol was paired with 2-octanol. These odorants are often used in bee conditioning experiments^8^. Sucrose or fructose (40%) was used as US.

During a learning trial, bees were stimulated with odorant for 3 s (CS+ or CS−). The bee antenna was touched with sugar solution 2 s after the beginning of odorant stimulation for the CS+ but not for the CS−. If bees showed PER after antenna stimulation with US they were fed 1 µL of the sugar solution used as US.

Memory retrieval tests were performed 1 h and 24 h after conditioning. During a retrieval test both CS+ and CS− were presented in a random order at 1 min intervals without US and we recorded whether bees showed PER. Bees were then fed with 5 µl of sucrose 40% to test for their motivation and bees non-responding by PER were discarded. Once fed, bees were kept at 28 °C in the dark with 70–80% relative humidity until the 24 h test, which was performed the same way.

### Data analysis

Statistics were computed using R Studio 1.1.423 and R3.4.0; α was set to 0.05.

Electrophysiological recordings were analyzed for each odorant using a mixed model; the factors were thymol treatment (0, 0.1 or 1 µg/µl), odorant concentrations (0%, 0.1%, 1%, 10% and 100%, treated as a repeated measurement within each bee, i.e. the factor bee was random) and their interaction. Sample sizes are reported in Fig. 1.

In behavioral experiments, there were 20 to 42 bees (average 30) for each combination odorant/thymol/sugar (Table 1). In each experiment the measure we report is the occurrence of PER to the odorant alone. Bees who didn’t show PER for US stimulations were discarded. As PER is binary data, we used generalized linear models to assess the effects of the three factors (sugar reward [two levels: sucrose or fructose], conditioned odorant [i.e. CS+: either one of 1-hexanol, 1-octanol, 2-hexanol or 2-octanol] and thymol treatment [three levels: 0, 0.1 or 1 µg per animal]) and of their interactions. Performance during retrieval tests was evaluated using logistic regressions. Performance during training was evaluated using generalized estimating equation (GEE), a type of logistic regression used for repeated measurements; in that case, we used trial as a repeated measurement within each bee (i.e. random factor) beside sugar, odorant and thymol factors.

To evaluate whether the different treatments affect odorant discrimination during the retrieval tests we performed McNemar’s test to compare within each combination odorant*sucrose*thymol the response rate between CS+ and CS−. McNemar’s test was also used to compare performance during the 4^th^ learning trial (the last one) and each of the two retrieval tests; in that case, Holm’s correction was used to correct for repeated measurements (Table 2).
